# The gesture imitation test in dementia with Lewy bodies and Alzheimer's disease dementia

**DOI:** 10.3389/fneur.2022.950730

**Published:** 2022-07-29

**Authors:** Xudong Li, Miaoxin Shen, Ziling Han, Jinsong Jiao, Xiaopeng Tong

**Affiliations:** ^1^Department of Cognitive Disorder, Beijing Tiantan Hospital, Capital Medical University, Beijing, China; ^2^China National Clinical Research Center for Neurological Diseases, Beijing, China; ^3^Department of Neurology, China-Japan Friendship Hospital, Beijing, China; ^4^Department of Microbiology and Immunology, Medical School, Xizang Minzu University, Xianyang, China

**Keywords:** dementia with lewy bodies, Alzheimer's disease dementia, gesture imitation, visuospatial function, neuropsychological test

## Abstract

**Objectives:**

Dementia with Lewy bodies (DLB) is the second most common type of neurodegenerative dementia following Alzheimer's disease dementia (ADD). This study investigated the diagnostic role of the gesture imitation test in detecting DLB and differentiating DLB from ADD.

**Methods:**

A total of 63 patients with DLB, 93 patients with ADD, and 88 healthy controls were included in this study. All participants were administered the gesture imitation test, the Mini-Mental State Examination (MMSE), the Montreal Cognitive Assessment (MoCA), the clock drawing test (CDT), and other neuropsychological tests.

**Results:**

The patients with DLB performed worse than the healthy controls in the global scores and on every item of the gesture imitation test (*p* < 0.001). The area under the curve (AUC) for the global scores was 0.889 (*p* < 0.001) in differentiating the DLB and control groups. Item 4 was a better discriminator, with a sensitivity of 79.37% and a specificity of 79.55%. The AUC for the global scores decreased to 0.593 and the difference was marginal (*p* = 0.079) in differentiating the DLB and ADD groups. The patients with DLB performed worse on Items 1 and 4 compared with the patients with ADD (*p* = 0.040, 0.004). The gesture imitation test was positively correlated with the scores of the MMSE (*r* = 0.355, *p* = 0.017), the MoCA (*r* = 0.382, *p* = 0.010), and the CDT (*r* = 0.407, *p* = 0.005) in patients with DLB.

**Conclusion:**

The gesture imitation test is an easy, rapid tool for detecting DLB and has a role in differentiating DLB from ADD, especially in Items 1 and 4.

## Introduction

Dementia with Lewy bodies (DLB) is the second most common type of neurodegenerative dementia following Alzheimer's disease dementia (ADD). The core clinical features of DLB include fluctuating cognition, recurrent visual hallucinations, REM sleep behavior disorder, and parkinsonism (1). There are several indicative biomarkers for DLB, including reduced dopamine transporter uptake in the basal ganglia demonstrated by SPECT or PET, abnormal ^123^iodine-MIBG myocardial scintigraphy, and polysomnographic confirmation of REM sleep without atonia. However, it is still difficult to diagnose DLB in the early stages because direct biomarkers of Lewy bodies are not yet available. Disproportionate attentional, executive function, and visual processing deficits relative to those in memory and naming, especially spatial and perceptual abnormalities, are typical manifestations in the early stage of DLB (1).

The gesture imitation test is a test that involves the imitation of meaningless gestures, which is considered a form of praxis and evaluates visuospatial and visuomotor abilities as individuals copy hand positions. The test is a useful tool for discriminating between patients with ADD, even patients with mild ADD, and healthy controls, according to our previous study (2). The test is not only correlated with the general neuropsychological tests and the Clinical Dementia Rating (CDR) but also correlated with the clock drawing test (CDT) and the pentagon part of the Mini-Mental State Examination (MMSE), which reflect impairment in visuospatial function. Because the standard neuropsychological tests are complex and time-consuming, the gesture imitation test is suitable for patients suspected of dementia in outpatient clinics.

The purpose of this study was to investigate the diagnostic role of the gesture imitation test in patients with mild and moderate DLB and the capacity of the test in differentiating DLB from ADD in memory clinics.

## Methods

### Subjects

A total of 243 subjects were recruited from the memory clinics between 2015 and 2019. All participants underwent routine assessments, including standardized history taking, physical and neurological examinations, necessary laboratory tests, and a CT or MRI scan. Of these patients, 93 (42 men and 51 women, mean age 77.55 ± 7.00 years, range 58–89 years) met the consensus criteria of NIA-AA (the National Institute on Aging and Alzheimer's Association workgroups) for probable ADD (3); 63 patients (44 men and 19 women, mean age 75.58 ± 7.56 years, range 58–86 years) were diagnosed with probable DLB according to the clinical criteria of the consortium (1); and 88 subjects were considered healthy controls, including 27 men and 61 women with a mean age of 71.20 ± 6.31 years. The inclusion criteria for the controls were as follows: (1) nearly normal cognitive function by inquiring informants; (2) a score of the MMSE equal to or above 26; (3) intact activities of daily living (ADL); and (4) a CDR score of 0 (4, 5). The healthy controls were excluded if they had a severe medical illness, a neurological disorder, a psychiatric disease, hearing or eyesight loss, and obvious abnormalities on a cranial CT or MRI scan. Participants who were prescribed psychiatric drugs were also excluded.

The objectives of the research study were explained to the participants and their families, and written informed consent was obtained. The research was approved by the Ethics Committee of the Beijing Tiantan Hospital.

### Clinical evaluations

The MMSE and Montreal Cognitive Assessment (MoCA) (6) were used for global cognitive screening. The scores of the MoCA were equal to or above 21 in all healthy controls and equal to or above 23 in 96.6% of controls. The CDT was used for visuospatial function and was scored by the Rouleau system (7). The ADL scores were also collected and analyzed (8). The behavioral and psychological symptoms of dementia were assessed by the neuropsychiatric inventory (NPI) (9). The CDR assessed the severity of dementia. The CDR scores were 1 or 2 in patients with ADD or DLB. Neuropsychological tests were administered and scored according to standard procedures.

### The gesture imitation test

The gesture imitation test was administered to all participants. The scorers rated the test during a neurological examination and were blinded to the cognitive test scores. The time required for test administration was ~1–2 min.

The test included the imitation of four meaningless gestures. The examiner sat face-to-face with the subject and instructed him/her to watch the examiner's hand gesture carefully and then imitate it. The instruction was repeated if necessary. The methods were administered according to those in our previous paper (2). Item 1 was performed with fingers III touching the thumb on flexion of the metacarpophalangeal joints with fingers II, IV, and V extended upward. Item 2 involved the intertwining of the left and right thumb-index circles. Item 3 intersected the left thumb and right little finger. Item 4 was performed with palms facing the body, then crossing both hands with fingers II–V extended upward and the two thumbs crossing each other. If the subject erred in any direction, with any finger, or with any intersection in 10 s, the score was 0; otherwise, 1 point was given. The maximum score for the gesture imitation test was 4.

### Statistical analysis

Statistical analyses were performed using SPSS, version 17.0 (SPSS Inc., USA). Data were expressed as the mean ± SD unless otherwise specified. One-way ANOVA was performed for quantitative variables among the control, DLB, and ADD groups, as well as for demographic data, the results of the neuropsychological tests, and the global scores of the gesture imitation test. The chi-square test was used to compare the differences between the qualitative variables among the three groups, including the sex ratio, handedness, and the percentage of the four gestures imitated perfectly. Receiver operating characteristic (ROC) analysis was performed to compare the diagnostic performance between the ADD and DLB groups and between the DLB and control groups. The sensitivity and specificity of every imitation subset were also assessed. Spearman's correlation coefficient (*r*) was used to evaluate the correlations between the gesture imitation scores and age, educational attainment, and neuropsychological test scores. All statistical tests were two-tailed, and *p* < 0.05 was used as the level of statistical significance.

## Results

The demographic and clinical data of the patients with ADD, the patients with DLB, and the healthy controls are summarized in Table 1.

**Table 1:** The demographic, neuropsychological data and gesture imitation test of the patients with DLB and ADD and the healthy controls.

**Items**	**DLB** **(*****n*** = **63)**	**ADD** **(*****n*** = **93)**	**Controls** **(*****n*** = **88)**	**F/**χ^2^	*p* **-Value**	**DLB vs. ADD**	**DLB vs. Controls**	**ADD vs. Controls**
**Age (years)**	75.58 ± 7.56	77.55 ± 7.00	71.20 ± 6.31	19.660	<0.001^*^	0.084	<0.001^*^	<0.001^*^
Gender (female percentage%)	30.2	54.8	69.3	22.724	<0.001^*^	0.002^*^	<0.001^*^	0.045
Education (years)	12.33 ± 4.67	11.17 ± 4.03	14.06 ± 2.77	16.526	<0.001^*^	0.307	0.033^*^	<0.001^*^
**Percentage of right handness (%)**	93.7	95.7	89.8	2.500	0.287			
Duration (years)	2.86 ± 2.13	2.86 ± 3.15	ND	2.709	0.102			
**Total scores of gesture imitation test**	2.09 ± 1.00	2.47 ± 1.09	3.57 ± 0.64	60.891	<0.001^*^	0.124	<0.001^*^	<0.001^*^
Item 1	0.71 ± 0.46	0.86 ± 0.35	1.00 ± 0.00	13.791	<0.001^*^	0.179	<0.001^*^	0.001^*^
Item 2	0.69 ± 0.47	0.59 ± 0.49	0.84 ± 0.37	7.172	0.001^*^	0.616	0.192	0.001^*^
Item 3	0.49 ± 0.51	0.59 ± 0.49	0.94 ± 0.24	30.038	<0.001^*^	0.586	<0.001^*^	<0.001^*^
Item 4	0.20 ± 0.41	0.43 ± 0.50	0.79 ± 0.41	34.115	<0.001^*^	0.015^*^	<0.001^*^	<0.001^*^
MMSE	19.38 ± 4.71	18.11 ± 4.00	28.33 ± 1.51	339.080	<0.001^*^	0.224	<0.001^*^	<0.001^*^
MoCA	13.51 ± 5.08	12.61 ± 4.07	26.27 ± 1.90	537.715	<0.001^*^	0.576	<0.001^*^	<0.001^*^
CDT	5.30 ± 3.27	6.07 ± 3.01	9.67 ± 0.71	110.147	<0.001^*^	0.370	<0.001^*^	<0.001^*^
ADL	38.18 ± 15.63	33.91 ± 8.26	21.22 ± 2.97	108.645	<0.001^*^	0.431	<0.001^*^	<0.001^*^
NPI	17.75 ± 15.69	16.55 ± 17.96	7.52 ± 9.63	12.193	<0.001^*^	0.981	0.008^*^	<0.001^*^
CDR	1.14 ± 0.59	1.25 ± 0.49	ND	1.512	0.221			

* *Statistically significant (p < 0.05)*.

*ADL, activity of daily living; CDT, clock drawing test; CDR, clinical dementia rating; MMSE, Mini-Mental State Examination; MoCA, Montreal Cognitive Assessment; ND, no data; NPI, neuropsychiatric inventory*.

The healthy controls were younger and more educated than the patients with DLB and ADD (*p* < 0.001), but there were no differences in age and educational attainment between the patients with DLB and patients with ADD (*p* > 0.05). There were more women in the groups of healthy controls and patients with ADD than in the group of patients with DLB (*p* < 0.001), whereas there were more women in the group of healthy controls than in the ADD group. The percentage of patients who were right-handed was similar among the three groups (*p* > 0.05).

The patients with DLB (19.38 ± 4.71, 13.51 ± 5.08, 5.30 ± 3.27, 38.18 ± 15.63, 17.75 ± 15.69) and patients with ADD (18.11 ± 4.00, 12.61 ± 4.07, 6.07 ± 3.01, 33.91 ± 8.26, 16.55 ± 17.96) performed worse than the healthy controls did (28.33 ± 1.51, 26.27 ± 1.90, 9.67 ± 0.71, 21.22 ± 2.97, 7.52 ± 9.63) on the MMSE, MoCA, CDT, ADL, and NPI, whereas the scores of the patients with DLB were similar to those of the patients with ADD (*p* > 0.05). In addition, there were no differences in duration and the CDR score between the two patient groups (*p* > 0.05).

**Figure 1:**
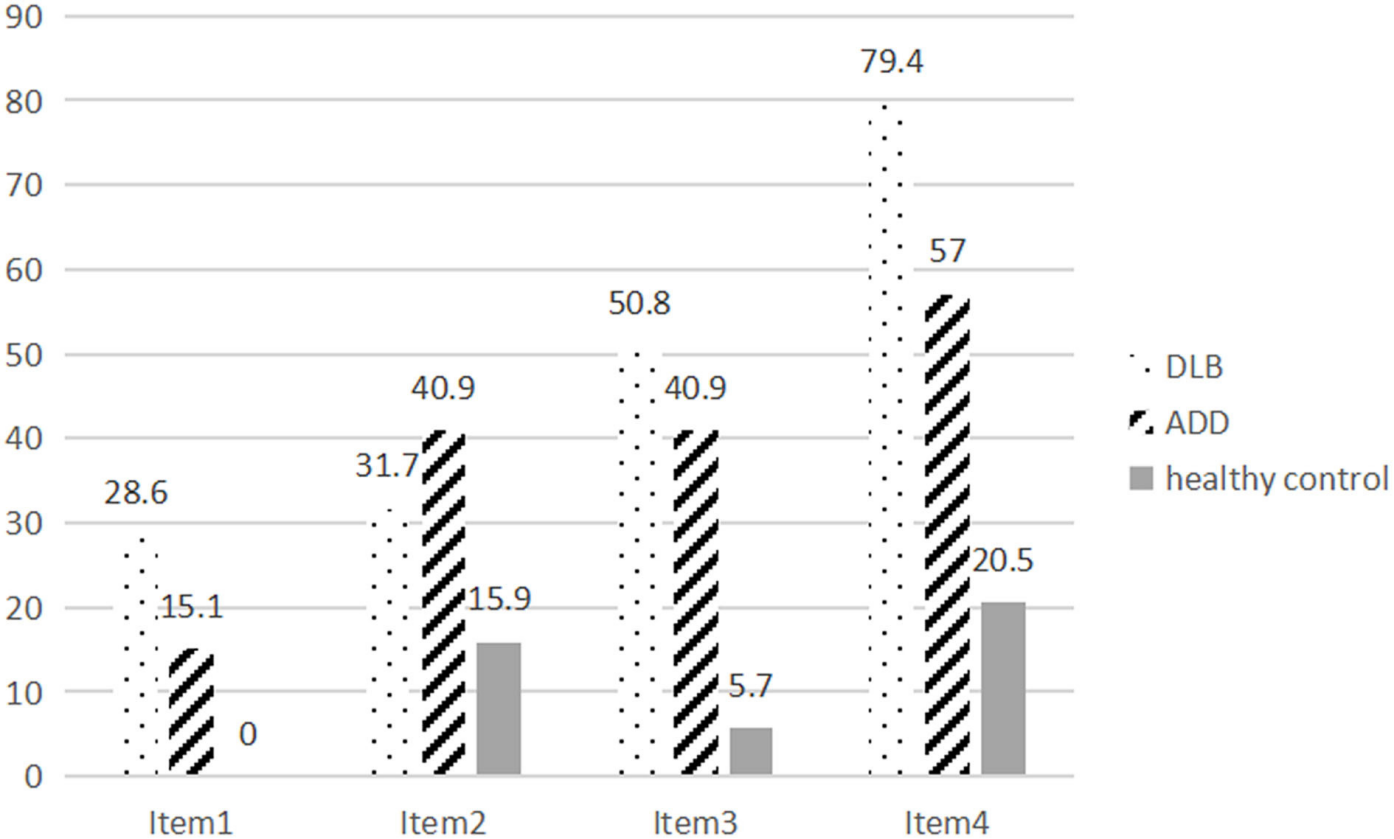
The error rate of Items 1–4 of the gesture imitation test in patients with DLB and ADD and healthy controls.

The global scores of the gesture imitation test in the controls (3.57 ± 0.64) were higher than those of the patients with ADD (2.47 ± 1.09) and DLB (2.09 ± 1.00; *p* < 0.001). The patients with DLB also performed worse than those with ADD did, but the difference was not significant (*p* = 0.124). The error rate of each item in the DLB and ADD groups was further analyzed (Figure 1). The error rate was higher in the DLB group (28.6, 31.7, 50.8, 79.4%) than in the healthy controls (0.00, 15.9, 5.7, 20.5%) on each item (*p* < 0.001, 0.022, <0.001, <0.001). The error rates were also higher in the ADD group (15.1, 40.9, 40.9, 57.0%) than in the healthy controls (*p* < 0.001, <0.001, <0.001, <0.001). The patients with DLB performed worse on Items 1 and 4 compared with the patients with ADD (*p* = 0.040, 0.004), but the error rate was similar on Items 2 and 3 between the patients with DLB and those with ADD (*p* = 0.248, 0.221). The mean scores of Items 1, 3, and 4 were lower in the DLB group than those in the healthy controls, whereas those of Items 1–4 in the ADD group were lower compared with those in the healthy controls. Only the scores of item 4 in the DLB group were lower than those in the ADD group (*p* = 0.015).

The area under the curve (AUC) for the global scores of the gesture imitation test in differentiating the ADD and control groups was 0.789 (CI = 0.723–0.856; *p* < 0.001), which was similar to that in our previous study. Among the items, Items 1, 2, and 3 had high specificity (100.00, 84.09, and 94.32%, respectively) and low sensitivity (15.05, 40.86, and 40.86, respectively). Item 4 had higher sensitivity, with a sensitivity of 56.99% and a specificity of 79.55%. The AUC for the global scores of the gesture imitation test increased to 0.889 (CI = 0.834–0.945; *p* < 0.001) in differentiating the DLB and control groups. Item 4 was also a better discriminator, with a sensitivity of 79.37% and a specificity of 79.55%. Items 1, 2, and 3 also had high specificity (100.00, 84.09, and 94.32%, respectively) and low sensitivity (28.57, 31.75, and 50.79%, respectively). The AUC for the global scores of the gesture imitation test decreased to 0.593 (CI = 0.495–0.691), and the difference was marginal (*p* = 0.079) in differentiating the DLB and ADD groups. Item 1 had high specificity, with a specificity of 84.94% and a sensitivity of 28.57%, and Item 4 had high sensitivity, with a sensitivity of 79.37% and a specificity of 43.01%.

In the DLB group, the gesture imitation test was positively correlated with the scores of the MMSE (*r* = 0.355, *p* = 0.017), the MoCA (*r* = 0.382, *p* = 0.010), the CDT (*r* = 0.407, *p* = 0.005), and negatively correlated with the ADL score (*r* = −0.500, *p* = 0.008). There was no relationship between the gesture imitation test and age, educational attainment, duration, and the NPI and CDR scores (*p* > 0.05).

## Discussion

This study investigated the capacity of the gesture imitation test to detect mild and moderate DLB and differentiate DLB from ADD in patients in our memory clinics. The test could discriminate between patients with DLB and healthy controls because the AUC was 0.889. The test could also detect patients with ADD, as the AUC was 0.789, which was similar to that in our previous study. Items 1 and 4 could differentiate patients with DLB from patients with ADD well, but the role of the total scores of the test was not obvious. The gesture imitation test scores were clearly correlated with the CDT scores, which reflect impairment in visuospatial function. The gesture imitation test scores also correlated with the general cognitive test and ADL scores.

There are relatively independent dorsal and ventral neural circuits that process different aspects of a visual scene, according to a recent theory (10, 11). The dorsal visual stream processes vision inputs for actions, whereas the ventral visual stream processes vision inputs for perception. A meta-analytic review involving individuals with neuropathologically confirmed ADD and DLB showed that the DLB pathology appears to cause a moderate but detectable reduction in visuospatial skills relative to that of ADD (12). Patients with DLB perform significantly worse than patients with ADD on tests with object and form discrimination that engage the ventral visual stream, including perceptual discrimination tasks. Patients with DLB also perform worse than patients with ADD on spatial tasks that engage the dorsal visual stream, such as figure copy, visual assembly, spatial matching, and motion perception (11–13).

Pentagon copying in the MMSE was significantly more impaired in patients with DLB than in those with ADD according to different score methods (14). Patients with DLB performed worse than patients with ADD on the total score and all parameters except the closing-in phenomenon according to a new qualitative scoring method for the pentagon copy test (QSPT) in the MMSE (15, 16). Patients who were confirmed to have DLB by biopsy also performed worse than patients with ADD on a qualitative measure of the number of angles produced in a drawing at the initial evaluation and showed a faster decline over time in the QSPT total score and qualitative scores for closure/opening and rotation of the drawing (17, 18). The visuospatial function assessed by Addenbrooke's Cognitive Examination-Revised had a faster deterioration in mild cognitive impairment-DLB (MCI-DLB) (19).

In the CDT and cube copying tasks, there was no consistent difference between the patients with LB and ADD (12), but some studies have reported that patients with DLB showed significantly poorer performance on these tasks (16). For the MoCA subset, the CDT, lower scores were reported in the patients with DLB than in the patients with ADD (20). We did not find any difference between the DLB and ADD groups in the CDT, with similar scores of the MMSE and the MoCA, indicating that common copying and drawing tasks were not sufficiently sensitive to discriminate DLB from ADD or that we needed a more complex scoring method or more complex tasks.

The patients with prodromal DLB (MCI-DLB) had lower scores in praxis (pantomime of tool use and imitation of meaningless gestures) and visuospatial abilities assessed with the number location subtest of the VOSP than the healthy controls did (21). There was no difference in visuoperceptual abilities between the two groups. Other studies have also reported that patients with MCI-DLB have a worse visuospatial function as measured by the CDT, cube analysis, and number location subtest of the VOSP compared with patients with MCI-AD (22, 23). Notably, 88% of patients with MCI-DLB initially presented with attention and/or visuospatial impairment (24). Poor performance in determining the number of angles in the QSPT may have an additional role in predicting early DLB (17). All of these studies suggest that visual abnormalities may be mainly involved in the dorsal visual stream in the prodromal stage of DLB.

Our gesture imitation test evaluates visuospatial and visuomotor abilities involving the dorsal visual stream, which provided a possibility of detecting DLB. Patients with DLB may have more obvious abnormalities in the gesture imitation test due to more severe visuospatial abnormalities, although patients with ADD performed poorly on it. This study demonstrated that Items 1 and 4 could differentiate patients with DLB from patients with ADD, while the DLB group had lower total scores on the test compared with the ADD group, but statistical significance was not achieved.

Our gesture imitation test included 4 items. Item 1 only involved complex finger gestures, Items 2 and 3 had finger and hand movements involving body midline crossing, and Item 4 had a more complex reversal of hand movements, which made the gesture sequential. Item 4 was the best discriminator in detecting ADD among all of the gestures in our previous study (2). Item 4 was also a better indicator for diagnosing DLB, and Item 1 had good specificity. Furthermore, Item 4 had good sensitivity for discriminating DLB from ADD because it involved changing the palm orientation from facing the subject to facing the examiner to make the test more complex, which may partly contribute to its impact on the improved sensitivity. Item 1 only involved complex finger gestures, not hand movements, which were simple compared with other items. So it had good specificity, not sensitivity. The difficulty of Items 2 and 3 was between Item 1 and 4, which could not differentiate DLB from ADD.

A few studies involving imitation tests reported similar results. The patients with DLB performed better than the patients with AD in the CDR 1 group and worse than those in the CDR 2 group with the Yamaguchi fox-pigeon imitation test (25). Another study reported that the imitation of bimanual gestures was impaired nonspecifically in approximately half of the patients with mild dementia, whereas the imitation of finger gestures was significantly more impaired in patients with early DLB than in those with AD (26). Further studies showed that unilateral finger imitation was more similar to constructional tasks and was impaired more often in patients with DLB than in patients with AD, while bimanual gesture imitation was a complex task involving visuospatial attention, executive function, and visuomotor control (27).

The main pathological finding of DLB is the coexistence of Lewy-related pathology and AD-type pathology. Poor baseline visuospatial performance was strongly associated with a rapid rate of cognitive decline in biopsy-confirmed patients with DLB but not in patients with AD. Patients with DLB with poor visuospatial skills had fewer neurofibrillary tangles and were more likely to experience visual hallucinations than those with better visuospatial skills (28, 29). Among the biopsy-confirmed DLB subjects further divided into groups by their Braak stage, the DLB-LB group performed worse than the DLB-AD group on tests of simple and complex visuospatial processing (visual reproduction copy, clock copy, and block design) (30). Another study reported that DLB subgroups grouped according to their Braak stage did not differ in the Hooper Visual Organization Test scores, although patients with DLB performed significantly worse than those with AD on the test (11). All of these findings suggest that disproportionate visuospatial deficits are not related to the concomitant AD pathology but might primarily reflect the Lewy body pathology.

Hypometabolism and decreased blood flow in the primary visual and visual association cortex of patients with DLB were found with positron emission tomography or single-photon emission computed tomography scanning, and these findings were consistent with the white matter spongiform changes and gliosis in the occipital region that occur independently of the amyloid pathology (1). Decreased activation was demonstrated in visual area V5/MT in response to the presentation of visual objects during functional MRI in patients with DLB (31). Visual hallucinations in DLB also correlated with lower glucose metabolism and weaker metabolic connectivity in the parietal-occipital cortex (32). Therefore, visuoperceptual and visuospatial functions that may be dependent upon these cortices were disproportionately impaired in patients with DLB.

All of these results have provided pathological and functional imaging evidence that the gesture imitation test can differentiate DLB from AD.

With the onset of the global COVID-19 pandemic, there has been an obvious rise in the use of remote assessment of individuals with cognitive impairment to replace face-to-face activities as social distancing measures preventing disease transmission, such as telephone calls or videoconferencing (33). Our imitation test is easy, rapid (requiring only 1 min), and suitable for administration in clinics. In addition, it is also suitable for remote assessment in the present period. Drawing and copying tasks require pencil and paper or have an exceedingly difficult scoring method, and other visual assemblies, spatial matching, and perceptual discrimination tasks need more complex instruments.

This study has some limitations. First, the participants were selected from our memory clinics, not from the community, which may lead to bias. Second, the healthy controls were younger and had higher educational attainment, and the DLB group included more men, which may also affect the results. But further correlation analysis showed there was no relationship between the gesture imitation test and age in the healthy control or DLB group (*p* > 0.05). Only a weak negative correlation was found between the gesture imitation test and age in the AD group (*r* = −0.214, *p* = 0.041). Education attainment did not affect the test in every group. More participants should be included in further studies to exclude the effect of age and education.

## Conclusion

The gesture imitation test could detect mild and moderate DLB, which correlated closely with the CDT, general cognitive tests, and ADL. The gesture imitation test could also differentiate DLB from ADD, especially Items 1 and 4. Thus, the gesture imitation test is an easy, rapid tool for detecting DLB in outpatient clinics or remote assessments. Of course, the test should be performed on a larger population to further determine its usefulness.

## Data availability statement

The raw data supporting the conclusions of this article will be made available by the authors, without undue reservation.

## Ethics statement

The studies involving human participants were reviewed and approved by Beijing Tiantan Hospital. The patients/participants provided their written informed consent to participate in this study.

## Author contributions

XL, JJ, and XT contributed to the conception and design of the study. XL analyzed and interpreted the data. XL and XT revised the manuscript. XL, MS, and ZH contributed to the participants' enrollment and the clinical assessments. MS wrote the first draft of the manuscript. All authors contributed to the article and approved the submitted version.

## Funding

This study was supported by Key Projects of Strategic International Cooperation in Scientific and Technological Innovation in the National Key Research and Development Program (2018YFE0203600), Key Area in Research and Development Program of Guangdong Province (2018B030336001), and personnel development funding projects of Beijing Dongcheng District (DCQYYRC-791-01-DR).

## Conflict of interest

The authors declare that the research was conducted in the absence of any commercial or financial relationships that could be construed as a potential conflict of interest.

## Publisher's note

All claims expressed in this article are solely those of the authors and do not necessarily represent those of their affiliated organizations, or those of the publisher, the editors and the reviewers. Any product that may be evaluated in this article, or claim that may be made by its manufacturer, is not guaranteed or endorsed by the publisher.
